# 

**Published:** 1887-03-26

**Authors:** 


### The Hospital.

**An Institution, Family, and Parochial Journal of Hospitals, Asylums, and all Agencies for the Care of the Sick, Criticism and News.**

SATURDAY, MARCH 26TH, 1887.

### Books, Good and Otherwise.

We have received the following for review :—

Churchill, J. & A.
- Alpine Winter in its Medical Aspects. By A. Tucker Wise, M.D., etc.
- Pulmonary Consumption. By James Weaver, M.D., etc.
- Medical Galvanism. By Herbert Tibbits, M.D.

Kegan Paul & Co.
- Serbelloni. By J. W. Gilbart-Smith.

Longmans & Co.
- Gray's Anatomy. 11th edition.
- Hip Disease in Childhood. By G. A. Wright.

Sampson Low & Co.
- Health for the People. By Andrew Wilson, F.R.S.E., etc., Editor of *Health.*

Whiting & Co
- Traveller's Medical Guide. By H. M. Stanley.

EXCHANGES.

Macmillan & Co.
- The Practitioner. Jan. and Feb., 1887.

### The Literary Prize.

A PRIZE of **Two Guineas** will be given every month for the best story or incident based upon hospital, or asylum, or student life, or any incident connected with the professions of medicine or nursing.

### Hospital Housekeeping.

FORMS and full particulars of the Quilter Prizes under this head (see page 355 for 19th Feb.) may be had on application to the Editor of THE HOSPITAL, Norfolk House. W.C. All competitive essays to be sent in by 30th April, 1887. Prizes, £10 10*s.* and £5 5*s.*

*** THE CHILDREN'S CORNER and AMUSEMENTS WITH PROFIT will be found on page vi of this week's issue.

### The In- and Out-Patients' Hospital Diary.

This Diary is so arranged as to make some portion of it appear every week, and the whole in each monthly part. It has been very difficult to compile, and corrections will at all times be gratefully received. It has been suggested that similar information about the larger Provincial and Scotch Hospitals would be useful to many readers. We should be glad to hear if this is felt to be the case.

### ORTHOPÆDIC CASES.

| Hospitals and Terms of Admission. | Physicians, Surgeons, and Medical Officers, with Days and Hours of Attendance. |
|---|---|
| City Orthopædic Hospital, 27, Hatton Garden, E.C. Sec., Mr. E. Dereuth. Admission free | *Surgeons*, Mr. E. J. Chance and House-Surgeon. M. Tu. Th. F. 2.30. New cases Tu. F. 2.30 |
| National Orthopædic Hospital for the Deformed, 234, Gt. Portland St., W. Sec., Mr. H. Canning. Free to poor; others by letter or payment | *In-Physician*, Dr. C. Beevor. *In- and Out-Surgeons*, Messrs. F. R. Fisher, M. Th. 2; W. J. Roeckel, Tu. F. 2; E. M. Little, W. 1 |
| Royal Orthopædic Hospital, 297, Oxford Street, W. Sec., Mr. B. Maskell. By Governor's letter | *Surgeons*, Messrs. B. E. Brodhurst, H. A. Reeves, C. Read, W. E. Balkwill, H. F. Baker. Daily, 1 |
| St. Bartholomew's Hospital, Smithfield, E.C. | *Surgeon*, Mr. Walsham, M. 2.30 |
| St. George's Hospital, Hyde Park Corner, S.W. | W. 2. *See* General Hospitals |

### PARALYSIS, EPILEPSY, AND DISEASES OF THE NERVOUS SYSTEM GENERALLY.

| | |
|---|---|
| Hospital for Diseases of the Nervous System, 32, Portland Terrace, Regent's Park Road, N.W. Sec., Mr. Howgrave Graham. By contributor's letter, or payment according to means, but free to necessitous poor | *In-Physicians*, Drs. J. Althaus, Th.; Hughes Bennett, M.; G. Ogilvie, Tu. Hour 2. *Out-Physicians*, Drs. Laycock, F.; W. H. Syers, W.; J. Althaus, Th.; G. Ogilvie, Tu.; H. Bennett, M. Hour, 2. *In- and Out-Surgeons*, Messrs. J. Bloxam, A. P. Gould |
| National Hospital for the Paralysed and Epileptic, Queen Square, Bloomsbury, W.C. Sec. and Director, Mr. B. B. Rawlings. Most of the beds are reserved free to the necessitous poor; the others are for patients who contribute a Guinea a week | *In-Physicians*, Drs. J. S. Ramskill, Tu.; C. B. Radcliffe, M.; J. H. Jackson, F.; T. Buzzard, W. Hour, 2.30. *In-Surgeons*, Messrs. W. Adams, M. 3; V. Horsley, F. 2.30. *Out-Physicians*, Drs. T. Buzzard, W., H. C. Bastian, Tu.; W. R. Gowers. M.; D. Ferrier, F. 2.30; J. A. Ormerod, Tu. W.; C. E. Beevor, M; F. Hour, 2.30 |
| National Hospital for Diseases of the Heart and Paralysis, 32, Soho Sq., W. Sec., Capt. F. Handley. In-patients require recommendation of Life Governor. | *In-Physicians*, Drs. V. Ambler and A. Duncan, M. W. Th. *In-Surgeon*, Mr. G. Bennett, Tu. *Out-Physicians*, Drs. V. Ambler, A. Duncan, R. Varley, J. Murray, M. 2, 7; Tu. 3; W 2, 8; Th. 2; F. 3. *Out-Surgeon*, Mr. G. Bennett, Tu. 5 |
| West End Hospital for Diseases of the Nervous System, 73, Welbeck St., W. Hon. Sec., Mr H A. Dowell. Children only as in-patients. Out-patients by letter or payment. | *In- and Out-Physicians*, Drs. H. Tibbits, M. Th. 1.30; Stretch-Dowse, Tu. W. 1.30; S. H. Armitage, W. Tu. F. 6. *In- and Out-Surgeon*, Mr. Benson |

### DISEASES OF THE SKIN.

| | |
|---|---|
| Charing Cross Hospital, West Strand, W.C. | *Physician*, Dr. Sangster, M. 2 |
| Guy's Hospital, St. Thomas Street, S.E. | *Physicians*, Drs. Pye-Smith, Hale-White, Tu. 12 |
| Hospital for Diseases of the Skin, 52, Stamford St., S.E. Sec., Mr. S. Hayman. Free to the necessitous; also by payment | *Physician*, Dr. F. J. Payne. *Surgeons*, Messrs. J. Hutchinson, Waren Tay, and Wyndham Cottle. (Out-patients seen M. W. 3, Tu. Th. F. 2) |
| King's College Hospital, Portugal Street, W.C. | *Physician*, Dr. Duffin, F. 1 |
| London Hospital, Whitechapel Road, E. | *Physician*, Dr. S. Mackenzie, Th. 9 |
| Middlesex Hospital, Berners Street, W. | *Physician*, Dr. R. Liveing, Tu. 4 |
| North-West London Hosp. 18, Kentish Town Rd., N.W. | *Physician*, Dr. J. Stowers, Tu. 1.30 |
| Paddington Green Children's Hospital, Paddington | *Physician*, Dr. T. Colcott Fox, S. 9.15 |
| St Bartholomew's Hospital, Smithfield, E.C. | *Surgeon*, Mr. H. Cripps, F. 1.30 |

### DISEASES OF THE SKIN.—*Continued.*

| Hospitals and Terms of Admission. | Physicians, Surgeons, and Medical Officers, with Days and Hours of Attendance. |
|---|---|
| St. George's Hospital. Hyde Park Corner, S.W. | *Physician*, Dr. Cavafy, W. 1.30 |
| St. John's Hospital for Diseases of the Skin, 45, Leicester Sq., W.C. Branch, Markham Square, Chelsea, S.W. Sec., Mr. St. Vincent Mercier. In-patients by letter; out-patients free or by small payment | *Physicians*, Drs. Robinson, Dow, Harries, Campbell, Bourns. Daily 2 and 7. Markham Sq. Branch, M. 10 and Th. 10 and 7. *Surgeon*, Mr. J. Milton, Tu. |
| St. Mary's Hospital, Cambridge Place, Paddington, W. | *Surgeon*, Mr. Morris, M. Th. 9.30 |
| St. Thomas's Hospital, Westminster Bridge Road, S.E. | *Physician*, Dr. Payne, W. 1.30 |
| University College Hospital, Gower Street, W.C. | *Physician*, Dr. Crocker, W. 1.30, S. 9.15 |
| Westminster Hospital, Broad Sanctuary, S.W. | *Physician*, Dr. T. C. Fox, W. 1.30 |

### STONE AND OTHER DISEASES OF URINARY ORGANS.

| | |
|---|---|
| London Hospital, Whitechapel Road, E. | Daily, 1 30. *See* General Hospitals |
| St. Peter's Hospital for Stone and Urinary Diseases, Henrietta Street, W.C. Sec., Mr. W. E. Scott. Admission free. Only women and children are seen on Fridays. Operation day, Wednesday | *In-Surgeons*, Messrs. W. J. Coulson, F. R. Heycock. *Out-Surgeons*, Messrs. E. H. Fenwick, M. 2, S. 4 to 7; F. S. Edwards, M. 5 to 7, Th. 2; Heycock, Tu. 2, W. 5 to 7, F. 2 |

### DISEASES AND IRREGULARITIES OF THE TEETH.

| | |
|---|---|
| Belgrave Hosp. for Children, 77 & 79 Gloucester St., Pimlico | *Surgeon*, Mr. G. W. Payne, W. 9 |
| Charing Cross Hospital, West Strand, W C. | *Surgeon*, Mr. Fairbank, M. W. F. 9.0 |
| Dental Hospital of London, Leicester Sq., W.C. Sec., Mr. J. F. Pink. Poor free. For special operation a Governor's letter is required | *Surgeons*, Messrs. Canton, Gregson, Hepburn, S. Bennett, Underwood, Woodhouse, Hern, Matheson, Parkinson, Read, Rogers, Truman. Daily, 9 to 11 |
| Evelina Hosp. for Sick Children, Southwark Brdg. Rd. | *Surgeon*, Mr. R. Denison Pedley, Tu. 9 |
| German Hospital, Dalston lane, Dalston, E. | *Surgeons*, Messrs. A. P. Reboul, C. West, Th. 10 to 11 |
| Great Northern Central Hospital, Caledonian Rd, N. | *Surgeon*, Mr. E. Keen, W. 1.30 |
| Guy's Hospital, St. Thomas Street, S.E. | *Surgeons*, Messrs. Moon, Pedley, Tu. Th. F. 12.30 |
| Hospital for Consumption, Brompton, S.W. | *Surgeon*, Mr. C. J. Noble, W., and when wanted |
| Hospital for Sick Children, Gt. Ormond Street, W.C. | *Surgeon*, Mr. Cartwright, W. 9 |
| King's College Hospital, Portugal Street, W.C. | *Surgeon*, Mr. Cartwright, Tu. F. 10 |
| London Hospital, Whitechapel Road, E. | *Surgeon*, Mr. A. W. Barrett, Tu. 9 |
| London Temperance Hosp., Hampstead Road, N.W. | *Surgeon*, Mr. Adolphus Alexander, M. 2 |
| Metropolitan Free Hospital, Gray's Inn Road, W.C. | *Surgeon*, Mr. G. Curnock, Tu. Th. S. 9 |
| Middlesex Hospital, Berners Street, W. | *Surgeons*, Messrs. Bennett, M. 9.30; W. 9; Hern, W. 9, F. 9.30 |
| National Dental Hospital, 149, Gt. Portland Street, W. Sec., Mr. A. G. Klugh. The poor, and all urgent cases admitted free; others by Subscriber's letter | *Surgeons*, Messrs. H. Weiss, W. Weiss, M.; A. Smith, G. Bradshaw, Tu.; G. A. Williams, M. Davis, W.; A. F. Canton, H. G. Read, Th.; T. Gaddes, W. S. Thomson, F.; H. Rose, W. R. Humby, S. Hours 9 to 11 |
| National Orthopædic Hospital, 234, Gt. Portland st, W. | *Surgeon*, Mr. H. Harris |

### THE IN- AND OUT-PATIENTS' HOSPITAL DIARY.

### DISEASES AND IRREGULARITIES OF THE TEETH.—*continued.*

| Hospitals and Terms of Admission. | Physicians, Surgeons, and Medical Officers, with Days and Hours of Attendance. |
|---|---|
| New Hospital for Women, 222, Marylebone rd., W. | *Surgeon*, Mr. H. Apperley |
| North-West London Hosp., 18, Kentish Town Rd., N.W. | *Surgeon*, Mr. W. A. Maggs, F. 9 |
| Paddington Green Children's Hospital, Paddington | *Surgeon*, Mr. C. Vincent Cotterell, W. 9 |
| Royal Free Hospital, Gray's Inn Road, W.C. | *Surgeon*, Mr. H. Harris, M. Th. |
| Royal Hospital for Children and Women, Waterloo Bridge Road, S.E. | *Surgeon*, Mr. Whitehouse, F. 1.30 |
| St. Bartholomew's Hospital, Smithfield, E.C. | *Surgeons*, Messrs. Ewbank, Tu.; Paterson, F.; Ackery, F.; Mackrett, Tu. Hour, 9 |
| St. George's Hospital, Hyde Park Corner, S.W. | *Surgeon*, Mr. Winterbottom, Tu. S. 9 |
| St. Mary's Hospital, Cambridge Place, Paddington | *Susgeon*, Mr. H. Hayward, W. S. 9.30 |
| St. Thomas's Hospital, Westminster Bridge Road, S.E. | *Surgeon*, Mr. Truman, Tu. F. 10 |
| University College Hospital, Gower Street, W.C. | *Surgeon*, Mr. S. J. Hutchinson, W. 9.30 |
| Victoria Hospital for Children, Chelsea, S.W. | *Surgeon*, Mr. F. Fox, S. 9.0 |
| West London Hospital, Hammersmith Road, W. | *Surgeon*, Mr. H. L. Albert, Tu. F. 9.30 |
| Westminster Hospital, Broad Sanctuary, S.W. | *Surgeons*, Messrs. J. Walker and M. A. Smale, W. S. 9.15 |

### THROAT DISEASES.

| | |
|---|---|
| Central London Throat and Ear Hospital, Gray's Inn Road, W.C. | *Surgeons. See* "Ear Cases." M. W. Th. S. 2.30; Tu. F. 5 |
| Great Northern Central Hospital, Caledonian Rd, N. | *Surgeon*, Mr. Spencer Watson, W. 2 |
| Guy's Hospital, St. Thomas Street, S.E. | *Surgeon*, Mr. Symonds, Th. 1 |
| Hospital for Diseases of the Throat and Chest, 32, Golden Sq. Hon. Sec., Mr. G. C. Witherby. Poor free; others by small payment | *Physicians*, Drs. Wolfenden, Tu. F. 2.30; Macdonald, Tu. F. 6.30; Bond, W. S. 2.30. *Surgeon*, Mr. T. Mark Hovell, M. Th. 2.30 |
| King's College Hospital, Portugal Street, W.C. | *Surgeon*, Mr. Cartwright, Tu. F. 10 |
| Middlesex Hospital, Berners Street, W. | *Surgeon*, Mr. Hensman, Tu. 9 |
| North West London Hospital, 18, Kentish Tn. Rd., N.W | *Surgeon*, Mr. Collier, M. 1.30 |
| St. Bartholomew's Hospital, Smithfield, E.C. | *Surgeon*, Mr. Butlin, F. 2.30 |
| St. George's Hospital, Hyde Park Corner, S.W. | *Surgeon*, Dr. Whipham, Th. 1.30 |
| St. Mary's Hospital, Cambridge Place, Paddington | *Surgeon*, Mr. A. T. Norton, M. Th. 9.30 |
| St. Thomas's Hospital Westminster Bridge Road, S.E. | *Physician*, Dr. Semon, Tu. F. 1.30 |
| University College Hospital, Gower Street, W.C. | *Physician*, Dr. Poore, Th. 1.30 |
| Westminster Hospital, Broad Sanctuary, S.W. | W. 9. *See* General Hospitals. |

### DISEASES OF WOMEN, (OBSTETRIC CASES, ETC.)

| | |
|---|---|
| Charing Cross Hospital, West Strand, W.C. | *Physicians*, Drs. J. W. Black, Amand Routh, Tu. F. 1.30 |
| Chelsea Hospital for Women, Fulham Road, S.W. Sec., Mr. J. H. Easterbrook. Payment from 10*s.* 6*d.* a week. Poor by Governor's letter. Out-patients, if without subscriber's letter, pay 6*d.* each attendance | *In-Physicians*, Drs. J. H. Aveling, M. Th.; A. W. Edis, Tu. F.; F. Barnes, W. S. Hour, 2.30. *Out-Physicians*, Drs. Dickinson and Mackeron, M.; W. Travers and G. Harper, Tu.; F. Jones and J. I. Parsons, W.; and H. T. Rutherford, F. Hour, 2 |

### DISEASES OF WOMEN, (OBSTETRIC CASES, ETC.)—*Continued.*

| Hospitals and Terms of Admission. | Physicians, Surgeons, and Medical Officers, with Days and Hours of Attendance. |
|---|---|
| East London Hospital for Children and Dispensary for Women, Shadwell, E. A Subscriber's letter is required, except in urgent cases | *Out-Physicians*, M. Tu. W. Th. F. 1 and 3; Tu. F. 9. *See* "Children's Diseases." |
| German Hospital, Dalston Lane, Dalston, E. | *Physician*, Dr. Rasch, M. Th. 9 |
| Great Northern Central Hospital, Caledonian Rd., N. | *Physician*, Dr. F. Barnes, W. 2 |
| Grosvenor Hospital for Women and Children, 3 and 4, Vincent Square, S.W. | *Out-Physicians and Surgeons*, Tu. W. Th. S. 2. *See* "Children's Diseases." |
| Guy's Hospital, St. Thomas Street, S.E. | *Physicians*, Drs. Galabin, Horrocks, M. Tu. F. 1.30 |
| Hospital of St. John and St. Elizabeth, 47, Gt. Ormond Street, W.C. For females suffering from long standing disease. | *Physicians*, Drs. Constable and Tegart (no out-patients) |
| Hospital for Women, 29 and 30, Soho Sq., W. Sec., Mr. David Cannon. In-patients admitted free by Governor's letter; also by payment. Out-patients free | *In-Physicians*, Drs. Carter, W. S. 2; R. T. Smith, Tu. F. 2; E. Holland, Tu. F. 9.30. *In-Surgeon*, Mr. H. A. Reeves, M. 4, Th. 2; *Out-Physicians*, Drs. R. T. Smith, F. 10; E. Holland, W. 10; Mansell-Moullin, M. Th. 11; Bedford Fenwick, Tu. 10; J. Oliver, S. 10. *Out-Surgeon*, Mr. S. Osborn, Th. 2 |
| King's College Hospital, Portugal Street, W.C. | *Physician*, Dr. Playfair, Tu. Th. S. 2 |
| London Hospital, Whitechapel Road, E. | *In-Physician*, Dr. Herman, Tu. F. 1.30. *Out-Physician*, Dr. Lewers, W. S. 1.30 |
| Metropolitan Free Hospital, Gray's Inn Road, W.C. | *Physician*, Dr. A. Venn, W. 2 |
| Middlesex Hospital, Berners Street, W. | *In-Physician*, Dr. Edis, Tu. F. 1.30. *Out-Physician*, Dr. Duncan, W. S. 1.30 |
| New Hospital for Women, 222, Marylebone Road, W. Hon. Sec., Miss C. Vincent. By subscriber's letter. Outpatients pay 6*d.* entrance fee, and 2*d.* each visit. | *In-Physicians*, Drs. Anderson, Atkins, Marshall, de la Cherois. *Out-Physicians*, Drs. Marshall, De la Cherois, Hitchcock. Daily, 1 to 3, and S 9 to 10. (All the physicians are women) |
| North-West London Hospital, 18, Kentish Tn. Rd., N.W | W. 1.30. *See* General Hospitals |
| Royal Free Hospital, Gray's Inn Road, W.C. | *In- and Out-Physician*, Dr. Hayes, Tu. S. 9 |
| Royal Hospital for Children and Women, Waterloo Bridge Road, S.E. Sec., Mr. R. G. Kestin. | *Out-Physicians*, Daily at 2. *Out-Surgeon*, W. 2. *See* "Children's Diseases." |
| St. Bartholomew's Hospital, Smithfield, E.C. | *In-Physician*, Dr. M. Duncan, Tu. Th. 2. *Out-Physician*, Dr. Godson, W. S. 9 |
| St. George's Hospital, Hyde Park Corner, S.W. | *Physician*, Dr. Champneys, Th. 1.30 |
| St. Mary's Hospital, Cambridge Place, Paddington, W. | *Physicians*, Drs. Meadows, Tu. F. 9.30; H. Jones, M. Th. 1.30 |
| St. Thomas's Hospital, Westminster Bridge Road, S.E. | *Physician*, Dr. Jervis, Tu. F. 2 |
| Samaritan Free Hospital for Women and Children, Lower Seymour Street, W. Branch, 1, Dorset Street, Manchester Square, W. Sec., Mr. Geo. Scudamore. Governor's letter or card is required if the disease of the applicant is *not* peculiar to her sex. Out-patients department (free) is at Edward's Mews, Duke Street, W. | *In-Physicians*, Drs. P. Boulton, M. Prickett, Amand Routh. Daily, 2. *In-Surgeons*, Messrs. G. G. Bantock, J. H. Thornton, W. A. Meredith. Daily, 9.30. *Out-Physicians*, Drs. M. Prickett, and Amand Routh, W. S. 1.30. *Out-Surgeons*, Messrs. W. A. Meredith, and J. D. Malcolm, M. Th.; A. H. G. Doran, and W. S. A. Griffith, Tu. F. Hour 1.30 |
| University College Hospital, Gower Street, W.C. | *Physicians*, Drs. G. Hewitt, M. Th. 1.30; J. Williams, Tu. F. 2 |
| West London Hospital, Hammersmith Road, W. | *Physicians*, Drs. A. Venn, Moullin, W. S. 2 |
| Westminster Hospital, Broad Sanctuary, S.W. | *Physicians*, Drs. Potter, Tu. F. 2; Grigg, Tu. F. 9 |

### THE CHILDREN'S CORNER.

**Thirteenth Competition.**

No. 67. GEOGRAPHICAL PUZZLE.

I'm a well-known town of England,
Scholastic fame I own ;
Take from me a single letter,
Then I'm a precious stone.

No. 68. HIDDEN WORD. I am a word of six letters. My 1, 2, 5, 6 is what we enter a room by ; my 1, 2, 4 is a very small mark ; my 4, 5 is a preposition.

No. 69. CONUNDRUM. When does night draw nigh ?

No. 70. BURIED POETS. Sovereign pontiff. a mixture of black and white ; a tall man ; arid, and a place for wild beasts ; a sibilant, what you write with, and the title of a knight ; articulate intelligible sounds and value or merit.

No. 71. CHARADE.

My *first* is won and never lost ;
Reversed, it's now before ye ;
My *next* reversed is red as blood
In veins of Whig or Tory ;
My *whole's* so wondrous strange, that I
Must candidly confess it,
Though you're ingenious, it will be
A wonder if you guess it.

No. 72. ARITHMETICAL QUESTION. Take the nine figures from 1 to 9, and arrange them in such a way that their addition will make exactly 100.

**Results of Eleventh Competition.**

No. 57. PUZZLE SENTENCE. The answer here is Charles Dickens (the £=l).

No. 58. CONUNDRUM. That which gives a cold, cures a cold, and pays the doctor is a draught (draft).

No. 59. SQUARE WORD.

G O L D
O D E R
L E N A
D R A Y

No. 60. ENIGMA. The answer intended here was a "Yard," but one or two other ingenious replies have been received.

No 61. FIGURE WORD. This=V+I+X+EN (two-thirds of t, e, n) or VIXEN.

*All right*—Alsie, A. C. K., Peeler, Dot, P.S., Skye, Mikado, Scrooge, Paul Methven, Agatha, Tim ; *Four right*—Isobel, Ellie, Sutton ; *Three right*—Florrie, Little Mary, Bismarck, Ariadne.

**Letters from Little Folk.**

The subject for next week will be "Holidays." Tell me how and where you spent them last summer, what you chiefly did, and so on. The letters about "Studies" have very much interested me, for they all display a love for learning. Here is a tiny note from Ellie, who will, I hope, obtain the prize for which she is competing :—

DEAR MR. EDITOR,—I am afraid I should take up too much of your time if I were to recount all my school studies, but I like arithmetic the most, and I am competing for a prize which my teacher has offered for the best arithmetician.

Yours faithfully, ELLIE, aged 11.

4', Burgoyne Road, Brixton.

Florrie, in a delightful letter, tells me that she is extremely fond of *all* studies, but her preference lies in the direction of Divinity. Physical Geography also takes her fancy, and likewise Arithmetic, save "that rule of three which puzzles me." Skye is going in for the Cambridge Local at Christmas, and is working away to get up 900 lines of Virgil. Her favourite subjects are Physical Geography, Grammar, and Latin, and she tells me she is very fond of French poetry. Isobel likes painting and drawing, and informs me she attends a School of Art, which has been newly opened at Lincoln. Mikado's tastes are quite musical. Here is a letter from her :—

DEAR MR. EDITOR,—I suppose music is a "school study." I am sure, if so, it is my favourite. I have learnt a sonata of Beethoven's, and I can play nearly all of it without the notes. Now I am learning a sonata by Steibelt. I like History also, and French. I do think the puzzles such fun. My sister is doing the others. I wonder whether either of us will get a prize. Perhaps I shall come and see you some day when I am in London. I should like to very much.

Yours truly, MIKADO, aged 13.

12, Upper Tichborne Street, Leicester.

Halford tells me he likes Latin and Geography best. Scrooge writes :—

DEAR MR. EDITOR,—I have not much to say about my school studies. as I have written on the same subject before. I do not like History and French, as History has so many dates to remember, and French is so hard to pronounce. I like Geography, Arithmetic, Algebra, Latin, Dictation, and Grammar very much. I think drawing is very nice sometimes.—I remain, yours truly, SCROOGE, aged 13.

7, Charles Street, Pendleton.

Latin, Euclid, Drawing, are the subjects which Tim prefers. Peeler tells me he would like to see the time when the dead languages are no longer taught, and their place taken by French, German, Geography, and Arithmetic. While I quite admit the importance of the latter subjects, and the neglect of Geography in most of our schools, I cannot say I should like to see the classics ostracised. Their acquisition forms an excellent mental training, while no one can pretend to be a student of English, unless he has a fair acquaintance with the two tongues which have so enormously enriched our own vocabulary.

**Letter Prize.**

One mark each : Peeler, Tim, Halford, Isobel, Skye, Florrie Scrooge, Mikado, Ellie.

To decide the Letter Prize next week (several competitors being equal) 5 marks will be given for the best letter, so that I hope all my little friends will make a *special* effort.

Answers must be addressed to the "PUZZLE EDITOR," THE HOSPITAL, Norfolk House, Norfolk Street, Strand, W.C., not later than Thursday next. The "Children's Corner" Coupon (see advertisement pages) must be enclosed.

The address and full Christian name and surname, and the age of the competitor, must be stated in all replies. A *nom de plume* may be added for the purposes of publication.

### AMUSEMENTS WITH PROFIT.

### PRIZE COMPETITIONS.

**Quarterly Prize Competition.**

Began 22nd January ; Ends 16th April.

**A Prize of Three Guineas**

will be awarded to the Competitor who shall have sent in the largest number of correct or best answers. No one will be permitted to win the Three Guinea Prize twice in any one year, but we shall award annually an additional

**Prize of Five Guineas**

to the competitor who shall have sent in the largest number of correct or best answers during the twelve months.

**No. 19. Double Acrostic.**

The cause of *these*, this Journal pleads ;
In Elis for its *games* renowned ;
Spill this, sir, it to *ill-luck* leads ;
'Twas once an *El Dorado* found ;
A town upon the *Ivel* stands ;
A *marshal* brave who Landau won;
'Twas Aphra Behn's *poetic name ;*
My *Euphues*, Scott has turned to fun.

(The initials and finals of the above acrostic give the title of a well-known movement for assisting our medical charities.)

**No. 20.—Mathematical Question.**

I had a certain number of oranges, and divided them among three persons. The first had half the oranges and half a one ; the second had half of the remainder and half a one ; and the third had half of what were then left and half a one. How many had I at first ? None were cut.

**Answers.**

No. 15. DOUBLE ACROSTIC.

| | | |
|---|---|---|
| H | avo | C |
| O | tag | O |
| S | overeig | N |
| P | ari | S |
| I | gla | U |
| T | eetotalis | M |
| A | llsop | P |
| L | igh | T |
| F | ol | I |
| O | gli | O |
| R | ui | N |

Correct answers have been received from Ostrich, Perseverance, Robe, Patient, Mater, Tabs. Boss, Mr. J. High, A. M. E., Peeler, Gretta, Ellice, Gip, Condorcet, K. M.

**No. 16. Mathematical Question.**

Two solutions are possible, viz., 5 larks, 4 wrens, and 12 sparrows, or 4 larks, 15 wrens, and 2 sparrows.

Correct answers have been received from Mater, Paddy, Tabs. Boss, Mr. J. High, A. M. E., Patient, K. M., Condorcet, Perseverance, Gip, Ellice, Peeler, Hexameter, Gretta, Ostrich, Robe.

**Notices to Correspondents.**

PERSEVERANCE.—Many thanks for yours of 14th.

A. M. E.—We are obliged for your suggestions, and will see what we can do.

FRED. C.—Answer to No. 9 not received from you.

Answers must be addressed to THE PRIZE EDITOR, AMUSEMENTS WITH PROFIT, THE HOSPITAL, Norfolk House, Norfolk Street, Strand, W.C. not later than the *first* post on Saturday next. The full name and address must in all cases be given, but a *nom de plume* may be added for publication. Only one side of the paper should be written on. The "Amusements with Profit" Coupon (see advertisement pages) must be enclosed with replies.

